# Impaired Glucose Tolerance in Adults with Duchenne and Becker Muscular Dystrophy

**DOI:** 10.3390/nu10121947

**Published:** 2018-12-07

**Authors:** Emma L. Bostock, Bryn T. Edwards, Matthew F. Jacques, Jake T.S. Pogson, Neil D. Reeves, Gladys L. Onambele-Pearson, Christopher I. Morse

**Affiliations:** 1 Research Centre for Musculoskeletal Science & Sports Medicine, Department of Exercise and Sport Science, Manchester Metropolitan University Cheshire, Crewe CW1 5DU, UK; matthew.jacques@stu.mmu.ac.uk (M.F.J.); jake.pogson@stu.mmu.ac.uk (J.T.S.P.); g.pearson@mmu.ac.uk (G.L.O.-P.); C.Morse@mmu.ac.uk (C.I.M.); 2 The Neuromuscular Centre, Winsford CW7 4EH, Cheshire, UK; bryn.edwards@nmcentre.com; 3 Research Centre for Musculoskeletal Science & Sports Medicine, School of Healthcare Science, Faculty of Science and Engineering, Manchester Metropolitan University, Manchester M1 5GD, UK; n.reeves@mmu.ac.uk

**Keywords:** Duchenne muscular dystrophy, Becker muscular dystrophy, diabetes, oral glucose tolerance test, body composition, muscle size

## Abstract

The aim of this study was to determine the response to an oral glucose tolerance test (OGTT) in adult males with Becker muscular dystrophy (BMD) and Duchenne muscular dystrophy (DMD), and to investigate whether body composition contributes to any variance in the glucose response. Twenty-eight adult males with dystrophinopathy (BMD, *n* = 13; DMD, *n* = 15) and 12 non-dystrophic controls, ingested 75 g oral anhydrous glucose solution. Fingertip capillary samples were assessed for glucose at 30-min intervals over 2-h post glucose ingestion. Fat free mass relative to body mass (FFM/BM) and body fat (BF%) was assessed using bioelectrical impedance. Vastus lateralis muscle anatomical cross sectional area (VL ACSA) was measured using B-mode ultrasonography. Blood glucose was higher in MD groups than control at 60, 90 and 120 min post ingestion of glucose. Compared to controls, FFM/BM and VL ACSA were lower in MD groups compared to controls (*p* < 0.001). Glucose tolerance values at 120 min were correlated with FFM/BM and BF% in the BMD group only. Our results suggest that glucose tolerance is impaired following OGTT in adult males with BMD and DMD. It is recommended that adults with BMD and DMD undertake routine glucose tolerance assessments to allow early detection of impaired glucose tolerance.

## 1. Introduction

Muscular dystrophy (MD) represents an umbrella term encompassing a group of myogenic disorders of varying degrees of severity. These conditions each present with specific genetic origins resulting in defects or absences within proteins of the dystrophin-sarcoglycan complex [1]. Both Duchenne (DMD) and Becker (BMD) muscular dystrophy share an identical genetic locus of impairment (Xp21) and subsequently a similar anatomical distribution of musculoskeletal weakness [2]. Each of these dystrophinopathies are characterised by either absent or reduced expression of the cytoskeletal protein dystrophin, resulting in progressive muscle degeneration with similar anatomical distribution but varied severity [2,3,4]. DMD, characterised by non-functioning dystrophin, is the most severe dystrophinopathy, with an estimated incidence of 3 in 100,000 boys [5,6]. BMD involves partially functioning dystrophin, and is therefore a milder yet more variable form of dystrophinopathy, with an incidence of 2 in 100,000 male births [5,6]. The nuances of diagnosis and the variability of the genetic defects of BMD and DMD are covered in detail elsewhere [7,8].

Although the presentation of clinical features such as atrophy and weakness is well described in children and teenagers with DMD, the presentation of comparative data from adults with dystrophinopathies and adults without MD remains limited [9,10,11,12]. With improved juvenile support in DMD, mortality has improved [13,14]; with the potential increase in adult lifespan, the study of co-morbidities associated with adults living with dystrophinopathies is essential. Recently we have produced daily calorific guidelines for adults with BMD [11], and through analysis of body composition and muscle mass it was apparent that broader dietary advice would be beneficial within this population. It is known from the study of other clinical conditions associated with a decline in muscle mass, that more than just functional impairments occur. For example, a higher fat mass and lower skeletal muscle mass is associated with insulin resistance in musculoskeletal ageing [15] and is associated with a risk of pre-diabetes/impaired glucose tolerance (IGT) within the general population [16]. An increased prevalence of risk factors for the development of diabetes is understandable given that skeletal muscle is essential as a glucose reservoir and as a target for insulin [17].

Despite previous reports of glucose tolerance and insulin resistance in children with BMD and DMD, at present there is no description of the blood glucose response to an oral glucose tolerance test (OGTT) in adults with DMD and BMD. Clinical guidelines for children with DMD suggest glucose assessment should be “considered” [8], potentially because children with DMD do not consistently show IGT [18,19,20]. It is likely that the progressive nature of DMD plays a role in this observation, as non-ambulatory teenagers with DMD show impaired glucose tolerance in response to OGTT, compared to children with DMD who were still ambulatory [19]. Furthermore, despite children with DMD presenting with normal OGTT, fasted hyperinsulinemia is known to be associated with a high body fat percentage in children with both BMD and DMD [21]; we have previously reported higher body fat percentage in adults with DMD and BMD [11,22]. Despite the progressive nature of the condition and the increasing adult lifespan, there are no reports of the blood glucose response to an OGTT in adults with dystrophinopathies. In addition, given the association between body composition (low skeletal muscle mass and high percentage body fat) and impaired glucose tolerance, it is currently not known whether body composition may contribute to any difference in the blood glucose response in adults with dystrophinopathies.

The aim of the present investigation was therefore twofold: (1) to investigate the response to an OGTT in adults with BMD and DMD compared to controls without MD; and (2) to determine whether alterations in body composition contributes to any variance in the glucose tolerance response in MD. It is hypothesised that people with MD will display an impaired response to oral glucose ingestion as a result of lower skeletal muscle mass and higher body fat percentage.

## 2. Materials and Methods

### 2.1. Participants

Twenty-eight men diagnosed with DMD (*n* = 15; age 24.6 ± 4.3 years, age range 19–34 years, height 1.69 ± 0.07 m, mass 71.5 ± 13.8 kg) or BMD (*n* = 13; age 43.7 ± 7.3 years, age range 33–55 years, height 1.76 ± 0.07 m, mass 96.5 ± 20.6 kg) and twelve healthy adult male controls (age 27.1 ± 7.4 years, age range 21–49 years, height 1.80 ± 0.08 m, mass 79.7 ± 11.9 kg) volunteered to participate in this study. Four of the participants with BMD were ambulatory (able to walk with or without an aid), nine were non-ambulatory (relied on the use of a wheelchair) and all participants with DMD were non-ambulatory. In the DMD group 6 out of the 15 (40%) participants reported to be currently taking corticosteroids (Prednisolone, *n* = 5; Deflazacort, *n* = 1, and have done so for over 10 years) and an additional participant reported previous use. Control participants were adult males who were low to moderately physically active based on current physical activity levels determined by the bone specific physical activity questionnaire (5.3 ± 3.0). Control participants were unrelated to the DMD and BMD participants, were otherwise healthy and reported as having no family history of diabetes. Due to the difference in age associated with presentation of the conditions, the participants could not be age-matched between groups, but glucose tolerance is not reported to be affected across the age-range used in this current study (19–55 years) [23]. All participants provided written informed consent before taking part in the study, which was approved by the local Ethics Committee of Manchester Metropolitan University. All procedures complied with the World Medical Association Declaration of Helsinki [24].

### 2.2. Procedures

All participants attended two testing sessions on separate days; the MD group were tested at The Neuromuscular Centre (Winsford, UK) and the control group were tested at the Manchester Metropolitan University. Aside from measures of mass and stature, all participants were tested using the same equipment. Visit 1 consisted of anthropometric and Bioelectrical Impedance measurements, followed by an ultrasound scan of the vastus lateralis muscle. Visit 2 was used to assess the glucose response to an OGTT. Participants attended both sessions in a fasted state.

### 2.3. Anthropometric Measurements

Stature and mass were measured using a wall-mounted stadiometer and digital scales in the control group. In the MD group, height was calculated as point to point (index finger, elbow, shoulder and across midline) span. In order to account for the known discrepancy between standing height and arm span measures, a correction was applied consistent with regression data from adult Caucasian males, the known error for making this correction is 3.5% [25]. MD participant height is presented as this corrected value. Participants in the MD group were weighed in a digital seated scales system (6875, Detecto, Webb City, MO, USA). The weight of shoes, splints, slings etc. were subtracted from the gross weight post weighing. Body mass index (BMI), a primary risk factor for type 2 diabetes in the general population [26,27], was calculated as body mass/height^2^.

### 2.4. Bioelectrical Impedance

Body composition measurements were calculated using Bioelectrical Impedance Analysis (BIA) (Body STAT, 1500); all participants were measured once while seated and were at least two-hours postprandial, having completed no exercise in the previous 12 h. Two proximal electrodes were placed between the styloid process of the right ulna and radius and between the medial and lateral malleoli of the right ankle. Two distal electrodes were placed on dorsal surfaces of metacarpals and metatarsals. Values of fat mass (kg), body fat%, fat free mass (FFM) and relative FFM (FFM relative to body mass (FFM/body mass)) were recorded for each participant. BIA has been shown to be valid and reliable in comparison to DEXA, with a Pearson Correlation Coefficient of r = 0.99 in adults of healthy weight, and r = 0.78 in an overweight population [28,29]. BIA has been recommended to assess nutritional status of people with DMD [30], and has previously been shown to be valid when assessing in a seated vs. a supine posture (between −2.3 and 2.4% difference between supine and seated measures using BIA) [31].

### 2.5. Vastus Lateralis Anatomical cross Sectional Area

Anatomical Cross Sectional Area (ACSA) of the Vastus Lateralis (VL) muscle was measured using real-time B-Mode ultrasonography. Resting ultrasound scans were taken with the participant in a seated position. A series of transverse plane scans (width of probe, 7.5-MHz linear array probe, MyLab^TM^ Twice, Esaote, Cambridge, UK) were digitally recorded at the level of 50% of the VL muscle length. VL length was measured from origin to insertion point, determined via an ultrasound scan in the sagittal plane. Care was taken to ensure minimal pressure was applied to the VL during scanning to avoid compression of the muscle. Strips of echo-absorptive tape (Transpore, 3M, St. Paul, MN, USA) were placed longitudinally from the medial to lateral border of the muscle, at approximately 3 cm intervals. These strips of tape were used as echo-absorptive markers that project a shadow onto the ultrasound image to provide a positional reference into the scanned structures. Individual images each consisting of two reference markers were collected using capturing software (Adobe Premier Elements, version 10, Adobe Systems, San Jose, CA, USA). The shadows from the echo-absorptive markers allowed the images to be aligned along with the contour of the muscle, and the entire VL ACSA to be recreated in a single image (Graphic Image Manipulation Program, GIMP Development). The ACSA was then measured using digitising software (ImageJ 1.45, National Institutes of Health, Bethesda, MD, USA). This method of ultrasound to measure ACSA has previously been reported as a valid (Intraclass correlation = 0.998, and 1.7% mean error) and reliable (Intraclass correlation = 0.999) measure in comparison to MRI [32]. 

### 2.6. Oral Glucose Tolerance Test

Consistent with the guidelines of the American Diabetes Association [33], after a 12-h overnight fast, participants ingested a commercially available glucose solution (Rapilose^®^ OGTT Solution, Penlan Healthcare Ltd., Tunbridge Wells, UK), delivering the equivalent of 75 g of anhydrous glucose [33]. Fingertip blood samples were obtained 10 min prior to glucose ingestion and then 30, 60, 90 and 120 min post ingestion. Glucose levels were measured using a hand held blood glucose meter (OneTouch Select^®^ Plus, Lifescan Europe, Zug, Switzerland; coefficient of variation (CV) = 2.52%) [34]. The OGTT was chosen, as the sensitivity of alternative approaches (e.g., HbA1C and fasted glucose tolerance testing) are in question, particularly when screening for pre-diabetic conditions. 

### 2.7. Physical Activity Questionnaires

Participants completed a bone specific physical activity questionnaire (BPAQ) [35]. The BPAQ data was analysed using freeware developed by Weeks and Beck, and is presented as the “Total” BPAQ, a combination of current and past physical activity [35]. Although not specifically designed for measurements in individuals with disability, the BPAQ questionnaire has previously been used in MD, is sensitive to the loss of ambulation in adults with dystrophinopathy and shows significant correlations with disability specific health questionnaires, such as the PASIPD [10,11,36]. Participants with MD also completed a disability specific physical activity questionnaire (PASIPD—Physical Activity Score for Individuals with Physical Disabilities) [36]. This includes a lower activity threshold and has subsequently been validated as a reliable outcome measure for physical activity in people with disability [37]. 

### 2.8. Statistics

IBM SPSS Statistics 23 software was used to analyse the data. All data are presented as mean ± SD. Age, relative FFM, BPAQ and PASIPD violated the parametric assumption of normal distribution (Shapiro-Wilk test, *p* < 0.05), all other variables were normally distributed. All variables showed homogeneity of variance (Levene’s test, *p* > 0.05) except for BMI, fat mass (kg), BPAQ and glucose area under the curve (AUC). Differences between the three groups (DMD, BMD and controls) were analysed using a one-way between groups ANOVA with Bonferroni corrected post hoc pairwise comparisons for homogenous values and Dunnett’s T3 corrected post hoc pairwise comparisons for non-homogenous variables. A three-way repeated measures ANOVA, with Bonferroni corrected post hoc pairwise comparisons, was used to compare between groups differences in absolute OGTT values at each time point. Variables that violated normal distribution were compared between groups using the Kruskal Wallis test, with post-hoc Mann-Whitney U pairwise LSD (least significant difference) comparisons, where appropriate.

Spearman’s correlations were carried out to highlight any relationships between specific variables. Statistically significant differences and/or associations were accepted at α ≤ 0.05. Study power (*β*) and effect size (pε^2^) are also reported.

## 3. Results

### 3.1. Anthropometry and Body Composition

Participant characteristics and anthropometric measurements are displayed in Table 1. Age, body mass and BMI were higher in the BMD group than the DMD (*p* < 0.001, *p* = 0.001 and *p* = 0.035, respectively) and control groups (*p* < 0.001, *p* = 0.03 and *p* = 0.014, respectively); there was no difference in age, body mass or BMI between the DMD and control group. The DMD group had lower height than the BMD and control groups (*p* = 0.02 and *p* = 0.001, respectively), with no difference between the BMD and control groups. Fat mass was higher in the BMD group than the DMD (*p* = 0.04) and control groups (*p* = 0.001), and fat mass was higher in the DMD group compared to control (*p* = 0.005). Body fat% was lower in the control group compared to the MD groups (*p* < 0.001). FFM was lower in the DMD group compared to the BMD and control groups (*p* = 0.03 and *p* < 0.001, respectively). Relative FFM (FFM relative to body mass (FFM/Body mass)), was lower in the BMD and DMD group compared to controls (*p* < 0.001) with no difference between MD groups. VL ACSA was bigger in the control group compared to the BMD and DMD groups (Table 1, *p* < 0.001).

### 3.2. Oral Glucose Tolerance Test

**Figure 1:**
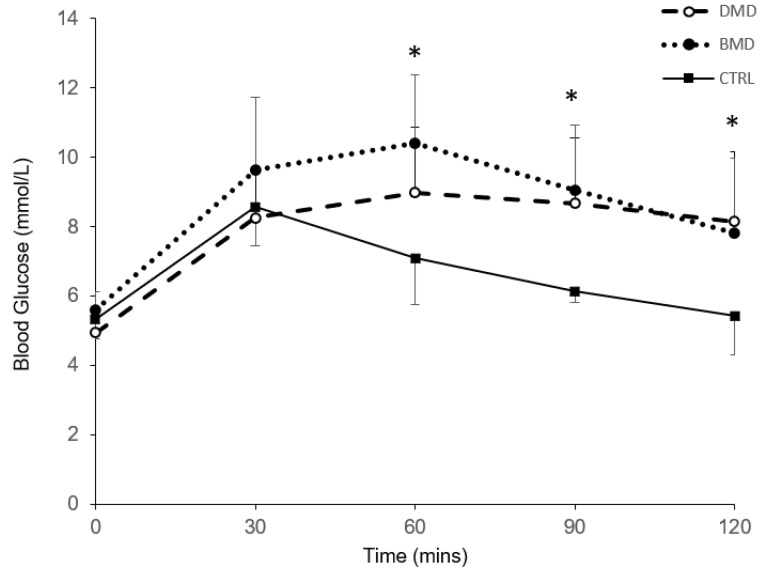
Blood glucose response to oral glucose intake. Blood glucose response to oral glucose intake in people with muscular dystrophy (MD) (Duchenne MD (DMD) dashed line, *n* = 15; Becker MD (BMD) dotted line, *n* = 13) and controls (solid line, *n* = 12). * denotes a significant difference between MD groups and controls (*p* < 0.05).

There was a significant effect of time (*p* < 0.001; pε^2^  =  0.557; *β*  =  1.000) and a significant time x group interaction (*p* < 0.001; pε^2^  =  0.223; *β*  =  0.979) on the blood glucose response (absolute values for each time point) to oral glucose ingestion (Figure 1). Blood glucose values were higher in the DMD and BMD groups compared to controls at 60, 90 and 120 min (*p* < 0.05, Figure 1), with no difference between DMD and BMD. 

Peak blood glucose values were significantly higher in the BMD group compared to controls (10.9 ± 1.7 mmol/L and 8.8 ± 1.0 mmol/L, respectively; *p =* 0.003), with no difference between DMD (9.9 ± 1.6 mmol/L) and either group. The AUC for the blood glucose values was higher in the DMD and BMD groups compared to controls (973 ± 144 mmol/L/120 min, 1073 ± 154 mmol/L/120 min and 815 ± 51 mmol/L/120 min, respectively; *p* < 0.05).

### 3.3. Physical Activity Questionnaires

Total BPAQ scores were significantly lower in the DMD and BMD groups compared to controls (Table 1, *p* < 0.001). PASIPD scores were not significantly different between groups (Table 1).

### 3.4. Correlations

Blood glucose 120 min post glucose ingestion and Glucose AUC were not correlated with any anthropometric measures (Table 2) in the DMD and control groups. Glucose 120 min post glucose ingestion correlated with body fat% and relative FFM in the BMD group (0.554, *p* = 0.049 and −0.557, *p* = 0.048, respectively). Physical activity scores from the BPAQ questionnaire correlated with Glucose AUC in the DMD group (0.713, *p* = 0.003). PASIPD scores were not found to correlate with any of the glucose tolerance values. Despite age being significantly higher in the BMD group than the DMD and control groups, age was not correlated with glucose tolerance values in any group (0.084, *p* = 0.78; 0.090, *p* = 0.75 and 0.007, *p* = 0.98, respectively).

## 4. Discussion

The main findings from the present study show impaired glucose tolerance following oral glucose ingestion in adult males with Becker and Duchenne MD. Our data show a significantly greater AUC for the blood glucose response to oral glucose ingestion in adults with DMD and BMD compared to controls. The blood glucose values two hours post glucose ingestion show that 53% of the DMD group, 46% of the BMD group and 0% of the control group met the ADA’s criteria for IGT (>7.8 mmol/L). Two of the DMD group met the ADA’s criteria for type II diabetes (>11.1 mmol/L). Our findings may highlight a potentially hidden problem of impaired glucose tolerance in the adults with BMD and DMD.

There are a number of potential mechanisms that can contribute to impaired glucose tolerance; we had originally hypothesised that lean mass or leg muscle area may be a factor. An association between relative FFM and blood glucose and between body fat percentage and blood glucose 2 h post OGTT was observed in the BMD group. As no other associations were observed between any anthropometric measure and glucose tolerance, the muscle reservoir hypothesis of glucose impairment [15,38] may not fully explain the impaired glucose tolerance in this population. It is possible that our observation of no relationship between VL ACSA or FFM and oral glucose tolerance, was due to the fact that what we have measured in terms of skeletal muscle mass may in fact overestimate the ‘true’ muscle contractile area in DMD and BMD participants due to the increased presence of intramuscular fat and connective tissue. Although alternative techniques exist for the assessment of contractile area in DMD and BMD these are usually in children, or more ambulant individuals [39,40,41,42,43]. The practicalities of scanning adults with DMD and non-ambulatory BMD with associated contractures and lack of mobility render this technique impossible with higher numbers of participants.

Although not reporting glucose tolerance data, previous observations in children with DMD and BMD aged 4–17 years show impaired insulin resistance, particularly in those with higher BMI and body fat [21]. A lower Insulin receptor affinity in children with DMD [18], particularly in erythrocytes [19], is likely to contribute to IGT observed in adults with BMD and DMD in the present study. Corticosteroids, often used in children with DMD [44], impair insulin-mediated glucose uptake by directly interfering with components of the insulin signalling cascade, such as glycogen synthase kinase-3, glycogen synthase and GLUT4 translocation [45]. In children with DMD, it is likely that the presentation of IGT could be linked to corticosteroids. Despite steroid-induced diabetes mellitus being reported in adults with other neurological conditions [46], in the present study IGT was no more prevalent in those using corticosteroids than non-users (2/6 currently taking corticosteroids had IGT). As only two participants of those classified as IGT, are currently taking corticosteroids, it would seem that the acute corticosteroid use is not a primary factor in the present BMD and DMD participants.

The discrepancy of significant IGT in ours compared to previous DMD data, that showed no significant glucose intolerance [19], can be attributed to the age of our participants (range 19–55 years) and the progressive nature of the condition. A distinction was previously made in the work of Freidenberg and Olefsky [19] such that, impaired glucose tolerance was reported only in those non-ambulatory participants (aged 14.7 ± 3.7 years), compared to the ambulatory participants (aged 8.9 ± 3.4 years) who showed no evidence of glucose intolerance. This would suggest condition severity plays a role in glucose tolerance impairments when comparing children to adults with DMD. It should be noted however, that the glucose tolerance impairments were similar between the ambulatory and non-ambulatory BMD participants, and between DMD and BMD participants in the present study. This similarity likely suggests that despite body composition being associated with glucose tolerance in BMD (consistent with non-MD previously [15,16]), the impairment is likely to relate to the insulin resistance identified above.

The prevalence of type 2 diabetes mellitus is rapidly increasing worldwide with known lifestyle aetiology being diet, obesity and physical inactivity [47,48,49]. The combination of a western diet (characterised by a high consumption of processed food, high-fat dairy products, sweets and desserts) with a low level of physical activity or obesity has been associated with a particularly high risk for type 2 diabetes [50]. BPAQ scores of physical activity levels were significantly lower in the MD groups than controls. Although not significantly correlated with glucose tolerance values, the lower level of physical activity and higher body fat percentage could be contributing factors to IGT in the current study. At present, we do not know whether diet has contributed to the observation of IGT in adults with DMD and BMD, but acknowledge that lifestyle factors could contribute. Further research is necessary to determine whether lifestyle, diet or the condition itself (or a combination of these factors) contributes to the observed IGT in the present BMD and DMD participants.

As mentioned above, previous observations from BMD and DMD, have shown no evidence of abnormal glucose tolerance that would lead to the classification of type 2 diabetes (a blood glucose level 2 h post OGTT of >200 mg/dL or >11.1 mmol/L) [18,19,20]. Based on 2 h glucose response, 2 of our 15 DMD participants would be classified as having type 2 diabetes, with 8 of our 15 DMD and 6 of our 13 BMD participants being classified as having IGT. There are a number of implications regarding these observations in terms of contraindications and management of unregulated glucose intolerance; it is well established that there are numerous cardiovascular, renal and neurological complications associated with microvascular impairments secondary to type 2 diabetes [51].

Regardless of the potential mechanisms that may contribute to the impaired glucose tolerance reported in the present study, the blood glucose levels at 120 min and greater glucose AUC has shown that adult males with dystrophinopathies are at risk of developing type 2 diabetes as their condition progresses with age. As can be observed from our participant demographics and recent studies, those with DMD are surviving well into adulthood. Guidelines for those with type 2 diabetes recommend non-pharmacological interventions such as diet and physical activity in the control of blood glucose [52]. Physical activity is unlikely to be effective or possible in most adults with DMD, and only a proportion of those with BMD. Nutritional management of impaired glucose tolerance, alongside pharmacological intervention may be required to manage blood glucose levels in adults with dystrophinopathies. In adults with BMD and DMD there are no nutritional guidelines regarding sugar intake, nor does there appear to be glucose tolerance tests commonly incorporated in regular clinical monitoring. Based on the observations in the present study, we strongly recommend that adults with DMD and BMD be offered glucose tolerance assessments to determine whether further treatment may be required related to impaired glucose tolerance. Our present data in adults also emphasises the need to develop dietary guidelines consistent with those for individuals with type 2 diabetes.

## Figures and Tables

**Table 1:** Participant characteristics and anthropometric measurements from adult males with (DMD and BMD) and without (Control) muscular dystrophy (MD).

	Control	DMD	BMD
N	12	15	13
Ambulatory	12	0	4
Current corticosteroid use (*n*)	0	6	0
Age (Years)	27.1 ± 7.4	24.6 ± 4.3	43.7 ± 7.3 *^,#^
Height (m)	1.80 ± 0.08	1.68 ± 0.07 *^,#^	1.76 ± 0.07
Body Mass (Kg)	79.7 ± 11.9	71.5 ± 13.8	96.5 ± 20.6 *^,#^
BMI (Kg/m^2^)	24.6 ± 2.5	25.2 ± 4.1	31.1 ± 6.7 *^,#^
Fat Mass (Kg)	13.9 ± 6.4	22.6 ± 6.2 *	36.9 ± 17.6 *^,#^
Body Fat%	16.4 ± 5.5	31.3 ± 3.8 *	35.4 ± 8.2 *
FFM (Kg)	65.7 ± 7.4	48.9 ± 8.6 *^,#^	59.5 ± 13.4
Relative FFM (%)	83.1 ± 5.5	68.7 ± 3.8 *	62.9 ± 12.7 *
VL ACSA (cm^2^)	32.2 ± 8.6	16.3 ± 9.5 *	16.9 ± 6.9 *
*n* with IGT	0	8	6
PASIPD	-	7.2 ± 7.1	12.9 ± 7.8
BPAQ	49.7 ± 48.0	5.9 ± 8.2 *	10.1 ± 10.2 *

Data are presented as mean ± SD. BMI, body mass index; FFM, fat free mass; VL ACSA, vastus lateralis anatomical cross sectional area; IGT, impaired glucose tolerance; PASIPD, Physical Activity Score for Individuals with Physical Disabilities; BPAQ, bone specific physical activity score. PASIPD scores are omitted for the control group, as the questionnaire is specific for persons with disability. * denotes a significant difference from control (*p* < 0.05). ^#^ denotes a significant difference between Becker muscular dystrophy (BMD) and Duchenne muscular dystrophy (DMD) (*p* < 0.05).

**Table 2:** Correlation analysis between anthropometric measurements, and markers of glucose uptake in males with (DMD and BMD) and without (Control) muscular dystrophy.

	Glucose 120 min			Glucose AUC		
	Control	DMD	BMD	Control	DMD	BMD
Body Mass	0.228	0.105	0.318	0.189	0.087	−0.107
BMI	0.193	0.086	0.394	0.305	0.046	0.017
Fat Mass	0.350	0.209	0.463	0.049	0.173	0.132
Body Fat%	0.322	0.148	0.554 *	−0.238	0.199	0.217
FFM	0.063	0.118	−0.268	0.224	0.041	−0.286
Relative FFM	−0.434	−0.148	−0.557 *	0.028	−0.199	−0.203
VL ACSA	0.434	−0.032	0.168	−0.224	0.279	−0.269

Data are presented as Spearman correlation coefficients. BMI, body mass index; FFM, fat free mass; VL ACSA, vastus lateralis anatomical cross sectional area. * denotes a significant correlation (*p* < 0.05).
